# Perceptions and Experiences of Women in Karachi, Pakistan Regarding Secondary Infertility: Results from a Community-Based Qualitative Study

**DOI:** 10.1155/2012/108756

**Published:** 2012-02-13

**Authors:** Neelofar Sami, Tazeen Saeed Ali

**Affiliations:** ^1^Department of Community Health Sciences, Aga Khan University, Karachi 74800, Pakistan; ^2^School of Nursing, Aga Khan University, Karachi 74800, Pakistan

## Abstract

*Background*. The prevalence of infertility in Pakistan is 22% with primary infertility at 4% and secondary infertility at 18%. This study explored perceptions and experiences of women in Karachi, Pakistan regarding the causes, treatment-seeking behavior for and consequences of secondary infertility. *Methods*. Focus group discussions and in-depth interviews with married women explored their perceptions and experiences for issues related to secondary infertility. *Results*. The knowledge of women about the causes and scientific treatment options for infertility was limited resulting in inclination for traditional unsafe health care. Infertility was stated to result in marital instability, stigmatization and abuse specially for women with no live child. *Conclusions*. Since infertility can have a serious effect on both the psychological well-being and the social status of women in Pakistan, effective interventions are the need of the day. There is a dire need for health education and counseling to be integrated into infertility management plans.

## 1. Introduction

Infertility is defined if a couple is unable to achieve pregnancy for at least one year of unprotected intercourse without using any contraceptives [1]. Globally, 10–15% of couples of reproductive age are infertile and prevalence varies from country to country. However, the trend of secondary infertility outnumbering the primary infertility is similar across all the developing countries [1, 2] with postabortal, puerperal [3, 4], and reproductive tract infections (RTIs) as the major causes. RTIs are either sexually transmitted [5] or result from infections introduced through medical procedures (during IUCD insertion, inducing an abortion or labor) [6]. Infertility has been viewed to be associated with increased psychological distress for the couples [7] with greater social pressures for women than men [8]. This is more marked for women with secondary infertility who either are unable to conceive again or face poor pregnancy outcomes such as abortions or stillbirths. Studies have shown that in these situations women not only are harassed by the family members but, face various forms of marital instabilities too [9, 10]. In Pakistan, the overall prevalence of infertility is 22% with primary infertility at 5% and secondary infertility at 18% [11]. There is a dearth of information about causes of infertility and health seeking behavior of infertile couples in the country.

A study was conducted to explore the perceptions and experiences of women regarding causes of, treatment-seeking behavior for, and consequences of secondary infertility. This paper describes the findings of the qualitative component.

## 2. Methods

### 2.1. Study Design

The qualitative research methods were used for better understanding of the contextual issues surrounding women's perceptions and experiences for causes and consequences of and health seeking behavior for secondary infertility. Focus Group Discussions (FGDs) and In-Depth Interviews (IDIs) were conducted to collect the information which gave a flavor of mixed methods.

### 2.2. Study Setting

The community-based study was conducted in urban areas of Karachi, Pakistan. The communities chosen were in the vicinity of selected infertility clinics. The data was collected from April to June 2006.

### 2.3. Study Participants

Purposive sampling methodology was used [12] to select currently married women aged 15–35 years for the study. FGDs were conducted with women having at least two live children to delve into the perceptions of fertile women about infertility. IDIs explored detailed experiences of women facing secondary infertility. The two methods demarcated the differences and similarities in perceptions and experiences. The local community-based health workers helped in identifying participants according to the selection criteria.

### 2.4. Process of Data Collection

Separate guidelines for FGDs and IDIs were developed in English, were translated into local language, and were pretested. The guidelines for FGDS and IDIs explored the participants' perspectives and experiences for causes (physical causes, menstrual irregularity, infections, etc.) and consequences of and health-seeking behavior for secondary infertility, respectively.

Ten FGDs and twenty IDIs were conducted. Each FGD had 6–10 participants. PI facilitated the discussions and interviews which were audio-taped. Notes were taken by note keepers. The data collection continued till the point of saturation when no new data was emerging [13].

### 2.5. Ethical Consideration

Before starting the data collection, ethical approval was obtained from the Ethical Review Committee of The Aga Khan University, Karachi. Written informed consent was obtained from all participants. The consent form was read for illiterate participants before having their thumb impressions. The consent form was signed by the PI, facilitator of the discussion/interviews, and by a witness.

### 2.6. Data Analysis

All the written and recorded materials were transcribed and translated into English. The data analysis started simultaneously with the data collection and was an iterative and continuous process. The analysis followed the analytic hierarchy, from data management to descriptive and explanatory account, as discussed by Spencer [12, 14]. 

**Figure 1:**
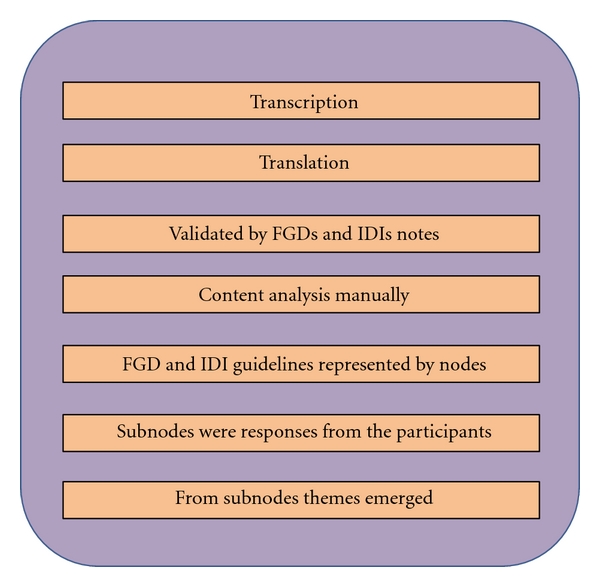
Analytical process of FGDs and IDIs.

Content analysis was done manually. The core FGD and interview guidelines were represented by nodes. From these responses (subnodes) and relevant themes were extracted. (Figure 1).

The identified themes were pooled together and integrated to generate concepts for study findings. The research team discussed and identified patterns, commonalities, and differences. During the second stage, the range of perceptions, views, and experiences within an identified theme were analyzed. Finally, links between experiences, behavior, perspectives, and characteristics of the participants were identified. To further ensure trustworthiness, triangulation of methods was undertaken by validating the findings from FGDs, IDIs, observation of group dynamics, and literature review. Results were discussed with qualitative researchers outside the research team.

## 3. Results

### 3.1. Sociodemographic Characteristics

Ten FGDs had total of 84 participants while IDIs were conducted with 20 women. (Table 1). The mean age of the participants of FGDs was 26.9 + 5.0 years and that of respondents of IDIs was 26.5 + 4.0 years.

The participants belonged to various ethnicities. Majority of the women were housewives and were either illiterate or had completed their primary education.

Important themes, which were highlighted through these discussions and interviews, are as follows (Table 2).

### 3.2. Causes of Infertility

The study participants gave a series of explanations for the causes of infertility categorized as follows.

#### 3.2.1. Magic and Spiritual Effects

The FGDs participants perceived magic and spiritual effects (local terminologies: *asaib and saya*) as main causes of secondary infertility among women. An evil spirit was held responsible which affects the women only and cannot influence a man. A participant mentioned that

“evil spirit lives in woman's body only as it affects the menstrual blood. Since men do not menstruate, it cannot live in their body”

(from FGD 5, woman aged 29, illiterate, mother of 3 children).

Some participants mentioned that the main cause of secondary infertility is a special action (*bandish*), done by spiritual healers, that shackles women's chances to conceive. 

The IDI respondents had similar views. While 12 women held evil spirits responsible, the remaining 8 reported menstrual irregularities, swelling/tumor, and wrong position of uterus as the cause. Interestingly, they believed that the physical causes were due to “*bandish*”. Those who had faced abortions or stillbirths believed that “*bandish*” “killed” their live pregnancies.

Regarding any anti-*bandish *remedies, the women believed that only special spiritual healers can offer such remedies against huge monetary compensation. It was interesting to note that both literate and illiterate women had similar beliefs.

#### 3.2.2. Unhygienic Practices during Menstruation, Intrapartum, and Postpartum Period

Regarding unhygienic practices during childbirth, menstruation, and postpartum period and resulting infections as causes of infertility, almost all FGDs participants negated and declared these as “dirty time periods and practices”. No one was aware that unhygienic practices during these phases could end up in infections and infertility.

“How can one have safe practices during dirty periods?”

A participant (FGD 2, aged 31 years, mother of four children, had primary education) asked.

IDI respondents were explored for their practices during menstruation, last childbirth, and puerperium. 18 respondents had home deliveries by untrained birth attendants and were not aware of hygienic care by them. 7 IDI respondents used intra-vaginal home-made medicines during last puerperium.

“These are good medicines for stopping bleeding and relieving pains”, a woman reported (IDI 3, illiterate, aged 25 and mother of 1 child).

Regarding menstrual practices, 17 IDI respondents reported the use of clothes, washed and dried in dark areas of the homes to hide these from others.

#### 3.2.3. Use of Modern Contraceptives

The FGDs participants declared that use of any contraceptive could result in infertility. Two participants mentioned infection following IUCD insertion can cause infertility. None of the IDI respondents reported using IUCD.

#### 3.2.4. Attempts for Terminating Pregnancy

The FGDs participants proclaimed an attempt for terminating pregnancy as a cause of secondary infertility and opined

“Termination of pregnancy is a sin, resulting infertility is the price for that”.

(FGD 3, woman aged 30 years, had 3 children and completed 9th class).

None of the IDI respondents reported any attempts for pregnancy termination. However, a woman mentioned “I consulted a TBA for a method for abortion but did not use any. My pregnancy aborted spontaneously. This is the punishment from God that I did not conceive again” (IDI 7, illiterate woman aged 35).

#### 3.2.5. Sexually Transmitted Infections (STIs)

Majority of the FGD participants did not know STIs as a cause of secondary infertility. A few participants thought that women with vaginal discharge and backache might face infertility as these cause weakness of the uterus.

“A weak uterus cannot retain a fetus and results in infertility”, a woman explained (FGD 7 woman aged 34 years, mother of 2 children, completed primary education). Women were not aware of any STIs among men.

5 IDI respondents reported history of foul smelling vaginal discharge and/or lower abdominal pain. However, none knew about any symptoms of STIs for their husbands.

#### 3.2.6. Causes in Men

All women from FGDs agreed that men cannot be responsible for secondary infertility as he proves to be “fine” by impregnating his wife. “If a man has erection power, he has power of reproducing. If woman has a stillbirth or does not conceive again then man cannot be blamed”, a FGD participant viewed (FGD 9, woman aged 29, illiterate, mother of 2 children).

IDIs respondents shared similar views. A respondent reported,

“My husband said that men cannot be infertile if woman had conceived once so there is no need for my investigation”

(IDI 12, woman aged 34 years, had completed primary education).

### 3.3. Treatment Seeking Behavior

The perceptions of FGDs participants and experiences of IDI respondents were similar that a woman with a live child waits for 2-3 years before seeking treatment but seeks treatment within 6 months if previous pregnancies ended in abortions/stillbirths. An IDI respondent sought treatment 2 months after her abortion.

#### 3.3.1. Selection of Health Care Providers

The FGD participants perceived that infertile women always consult physicians and gynecologists.

“This is a modern time and infertile women consult doctors only. In villages, women still opt for traditional healers,”

a woman opined (FGD 8, woman aged 34 years, mother of 3, and completed primary education).

All women agreed that if infertility is due to “*bandish*”, then woman should consult a spiritual healer.

The experiences of IDI respondents were different and physicians were not their first choice. Women consulted traditional healers (Traditional Birth Attendants (TBAs), *hakeems *(*a type of traditional healer*),* and homeopathics*) and spiritual/religious before a physician.

The infertile women declared TBAs to be the favorite providers for infertility as they do not advise for any investigations and the prescribed traditional medicines are inexpensive and free of side effects. They mentioned that treatment by physicians/gynecologist is a long process with a series of investigations and treatment. An infertile woman complained

“Husbands do not agree for investigations and physicians do not treat the wife alone so women stop consulting gynecologists and go to TBAs.”

Infertile women sought treatment from spiritual healers too. Two women consulted religious persons (*Pir Sahib) *to get a sacred locket (*taweez*) for tying around lower abdomen, that is, the place of uterus. Three women visited the tomb of some saint every Thursday night for consecutive 7 weeks. Other women reported the use of sacred water with herbs for drinking and/or vaginal douching.

### 3.4. Consequences of Secondary Infertility

The consequences of secondary infertility are discussed under the following headings.

#### 3.4.1. Social Consequences

The FGD participants agreed that woman has to bear the brunt of infertility and infertile women get threats for divorces, ejection from homes, and husbands' remarrying. All IDI respondents mentioned that they were being blamed for infertility and were threatened for husbands' second marriages. However, the negative attitude was less marked for women with a live son.

A woman with three stillbirths complained that at the occasions of marriages, she is kept away from the bride as she could transmit the “bad omen” to the bride.

Almost all infertile women with one live child, though thanked God for that blessing, felt that in Pakistani society having one child is equally a stressful. They said that their only child questions for not having any siblings. Two infertile women specifically talked about the high hopes of their families from the only child and feared immense pressure for him/her.

#### 3.4.2. Psychological Consequences

The FGDs participants mentioned depression, unhappiness, and dejection being prevalent among infertile women. Since men are not infertile, the participants assumed that they do not suffer as “he has always a chance to marry again”, a participant (FGD 1, woman aged 29, mother of 5 children) thought.

The infertile women agreed to be desperate and anxious for childbearing and lack of support from families/husbands and society's negative attitude instigates the thoughts for committing suicide. An infertile woman (IDI 6 aged 32 years) reported such attempt once.

#### 3.4.3. Economic Consequences

The perceptions and experiences for economical consequences were different. The FGD participants thought that one can pay “infinitely” to have a child. The infertile women found investigations and treatment for infertility to be expensive, and sometimes, unaffordable. One reason for opting spiritual/traditional remedies was their low cost.

### 3.5. Adoption as a Coping Mechanism

The FGD participants perceived that women with secondary infertility do not opt for adoption as she has been pregnant before and is optimistic to conceive again. Overall, participants felt that an adopted child is usually not accepted in a community. Majority of the IDIs respondents favored adoption but reported resistance by husbands and in-laws. The infertile women declared that their in-laws would support husband's remarrying over an adoption.

## 4. Discussion

The results of the qualitative component of the study have brought up various important conclusions. However, these could not be generalized as the respondents were selected purposively. Generally, in exploratory study associations can only be suggested and not demonstrated.

Infertility, whether primary or secondary, is labeled as a “disease” of woman and most of the time men are never investigated and treated. This is similar to the findings of other studies [15, 16].

Generally there was a lack of awareness regarding the causes of secondary infertility. Women were not aware of and rather negated any role of unhygienic practices ending up in puerperal and postabortal infections, PID, and infertility. Results from studies conducted in Africa, Bangladesh, and India have revealed unsafe practices as risk factors for infections, PID, and infertility [17–19]. This is particularly alarming in Pakistan where TBAs conduct majority of the deliveries [20] and unsafe abortions are common [21]. Such evidences are indicators for unhygienic practices.

Our study revealed that women's beliefs about superstitious causes of infertility influenced their health seeking behavior too. The studies conducted in Bangladesh and Nigeria have shown similar findings where the cause of infertility is assumed to be due to effects of bad evils resulting in seeking care from spiritual healers [22–24]. However, none of the studies have revealed if some strategies have ever targeted towards such beliefs to modify the behaviors.

The study has shown that though infertile women consult any category of providers, traditional untrained providers were a preferred choice for them. Other studies from Pakistan, India, and Nigeria have shown that traditional healers form quite a popular set of service providers for infertile women [16, 25]. Since the efficacy of both traditional and spiritual treatment has not been proven yet, approaching these healers causes delay in receiving the cause-based scientific management or could possibly be associated with worsening of infertility.

Regarding its consequences, the study has clearly shown that secondary infertility not only socially stigmatizes the women but also challenges their marital stability. Biological motherhood has always being viewed by society as an obligatory part of such relationships [26, 27].

The study has explored adoption as a coping mechanism for infertility. In the developed world, adoption has always been a choice for infertile couples [28]. Almost half of the women with secondary infertility in our study were willing to but could not adopt a child due to the resistance from the husband's family. In Pakistan, child adoption needs to be fully explored for infertile couples as there could be resistance in the families and communities due to perceived religious reasons [29] and fear of disloyalty by the child and claim by the biological parents.

## 5. Policy Implications

In the light of findings of the study, various policy and advocacy initiatives are required to address the issue of secondary infertility. There is a need for health education of the community for the correct knowledge of causes of secondary infertility, appropriate time for health seeking, and role of hygienic practices during menstruation, intrapartum, and postpartum periods so that problem could be prevented all together. Infertile couples spend substantial time and money for obtaining healthcare. Seeking traditional and spiritual remedies may result in certain complications due to their nonscientific use and causes delay in getting modern health care. Reproductive health programs should address these issues by training the health professionals at primary health care level. Additionally, it is important for health professionals to realize the stress and anxiety of infertile couples caused by persistent infertility and by undergoing complex investigations and treatment. The attending physician should be trained to give appropriate counseling and support to such couples. In Pakistan, further research in context of infertility in general and secondary infertility in particular would be an important step towards highlighting the extent of the problem and consequences. This would not only help in documenting the preventable causes and so help in precluding and decreasing the prevalence of the problem but would also address the issues related to psychological well-being and the social status of women in Pakistan.

## Figures and Tables

**Table 1:** Sociodemographic characteristics of women participants of FGDs and IDIs.

	Participants of FGDs (*n* = 84)		Respondents of IDs (*n* = 20)	
	*n*	%	*n*	%
Current age (years)				
<20 21–25 26–30 31–35	8 24 28 24	9.5 28.5 33.3 28.5	0 8 8 4	0.0 40.0 40.0 20.0
Ethnicity				
Urdu speaking Pathan Punjabi Sindhi	28 18 18 20	33.3 21.4 21.4 23.8	10 4 2 4	50.0 20.0 10.0 20.0
Respondent's educational qualification				
Illiterate Primary Secondary and +	34 30 20	40.4 35.7 23.8	8 8 4	40.0 40.0 20.0
Husband's education qualification				
Illiterate Primary Secondary and +	20 36 28	23.8 42.8 33.3	2 6 12	10.0 30.0 60.0
Respondent's occupational status				
Housewives Employed	58 26	69.0 31.0	16 4	80.0 20.0
Husband's occupational status				
unemployed Employed	6 78	7.2 92.8	0 20	0.0 100.0

**Table 2:** Common themes from analysis of FGDs and IDIs.

(1)	Causes of secondary infertility
	(i) Magic and spiritual effects (ii) Unhygienic practices during menstruation, intrapartum, and postpartum period (iii) Contraceptive use (iv) Termination of pregnancy (v) Sexually transmitted infections (vi) Causes in Men
(2)	Treatment seeking behavior
	(i) Time period before seeking care (ii)Selection of health care provider
(3)	Consequences
	(i) Social consequences (ii)Psychological consequences (iii) Economic consequences
(4)	Adoption as a coping mechanism
